# Probing formation of cargo/importin-α transport complexes in plant cells using a pathogen effector

**DOI:** 10.1111/tpj.12691

**Published:** 2014-11-17

**Authors:** Lennart Wirthmueller, Charlotte Roth, Georgina Fabro, Marie-Cécile Caillaud, Ghanasyam Rallapalli, Shuta Asai, Jan Sklenar, Alexandra M E Jones, Marcel Wiermer, Jonathan D G Jones, Mark J Banfield

**Affiliations:** 1 The Sainsbury LaboratoryNorwich Research Park, Norwich, NR4 7UH, UK; 2 Department of Biological Chemistry, John Innes CentreNorwich Research Park, Norwich, NR4 7UH, UK; 3 Department of Plant Cell Biology, Georg-August-UniversityJulia-Lermontowa-Weg 3, 37077, Goettingen, Germany

**Keywords:** importin-α, nucleo-cytoplasmic transport, nuclear localization sequence, oomycete effector, plant innate immunity, *Hyaloperonospora arabidopsidis*, *Arabidopsis thaliana*

## Abstract

Importin-αs are essential adapter proteins that recruit cytoplasmic proteins destined for active nuclear import to the nuclear transport machinery. Cargo proteins interact with the importin-α armadillo repeat domain via nuclear localization sequences (NLSs), short amino acids motifs enriched in Lys and Arg residues. Plant genomes typically encode several importin-α paralogs that can have both specific and partially redundant functions. Although some cargos are preferentially imported by a distinct importin-α it remains unknown how this specificity is generated and to what extent cargos compete for binding to nuclear transport receptors. Here we report that the effector protein HaRxL106 from the oomycete pathogen *Hyaloperonospora arabidopsidis* co-opts the host cell's nuclear import machinery. We use HaRxL106 as a probe to determine redundant and specific functions of *importin-*α paralogs from *Arabidopsis thaliana*. A crystal structure of the importin-α3/MOS6 armadillo repeat domain suggests that five of the six Arabidopsis importin-αs expressed in rosette leaves have an almost identical NLS-binding site. Comparison of the importin-α binding affinities of HaRxL106 and other cargos *in vitro* and in plant cells suggests that relatively small affinity differences *in vitro* affect the rate of transport complex formation *in vivo*. Our results suggest that cargo affinity for importin-α, sequence variation at the importin-α NLS-binding sites and tissue-specific expression levels of importin-αs determine formation of cargo/importin-α transport complexes in plant cells.

## Introduction

In eukaryotic cells the nuclear envelope acts as a selective barrier separating nuclear from cytoplasmic processes. Coordination of nuclear and cytoplasmic events is mediated by nuclear pore complexes (NPCs) that span the nuclear envelope. Low-molecular-weight compounds such as solutes and proteins with a molecular weight of <40–60 kDa can traverse NPCs by passive diffusion (Stewart, 2007; Wang and Brattain, 2007). Proteins of higher molecular weight rely on nuclear transport receptors (NTRs) for passage through NPCs. Notably, many nuclear proteins of molecular weight below 40–60 kDa, such as several transcription factors, are also imported by NTRs, presumably ensuring more efficient nuclear import compared with passive diffusion (Ballesteros *et al*., 2001; Krebs *et al*., 2010). NTRs of the importin-α/β class are conserved from yeast to plant cells and transport many distinct cargo proteins into the nucleus. Importin-αs act as adapter proteins. The importin-α armadillo repeat domain binds to nuclear localization sequences (NLSs) of cargo proteins whilst an N-terminal α-helix makes direct contact to importin-β and is therefore called the importin-β-binding (IBB) domain (Cook *et al*., 2007). The IBB domain contains a sequence related to bipartite NLSs and, in the absence of importin-β, the IBB domain competes with NLS-cargos for binding to the armadillo repeat domain. On the cytoplasmic side of the NPC, binding of the IBB domain to importin-β negates this auto-inhibitory effect of the IBB domain and therefore facilitates cargo binding to importin-α (Kobe, 1999; Harreman *et al*., 2003). Active transport of the ternary importin-α/β/cargo complex through the NPC is mediated by direct interactions between importin-β and Phe/Gly-repeat nucleoporin proteins that line the inner side of the NPC (Terry and Wente, 2009). On the nucleoplasmic side of the NPC the ternary complex is destabilized by binding of the GTP-bound form of the small GTPase Ran to importin-β, resulting in dissociation of the IBB domain from importin-β. This re-establishes the auto-inhibitory effect of the IBB domain on cargo binding and leads to release of cargo proteins on the nucleoplasmic side of the NPC (Görlich *et al*., 1996; Moroianu *et al*., 1996; Harreman *et al*., 2003).

Nuclear import rates in yeast correlate with formation of the importin-α/β/cargo ternary complex in the cytoplasm (Hodel *et al*., 2006; Timney *et al*., 2006). Thus, nuclear import kinetics are influenced by the cytoplasmic concentrations of both cargo proteins and NTRs, as well as the affinity of a particular cargo NLS for the NTR. The best characterized NLSs are Lys/Arg-rich sequence motifs that fall into two subgroups, monopartite NLSs with the consensus sequence (K[K/R]X]K/R]) and bipartite NLSs with two clusters of basic residues separated by a linker sequence ([K/R][K/R]X_10–12_[K/R]_3/5_) (Chang *et al*., 2012; Marfori *et al*., 2012). The importin-α armadillo repeats form two NLS-binding sites on the concave side of the protein, referred to as ‘major’ and ‘minor’ binding site. Whereas bipartite NLSs make contact to both binding sites, monopartite NLSs bind to either the major or the minor site (Marfori *et al*., 2011; Chang *et al*., 2013).

Adapted plant pathogens suppress host defences by translocating effector proteins into plant cells (Dou and Zhou, 2012; Petre and Kamoun, 2014). Several effectors that manipulate nuclear processes have evolved NLSs and co-opt the host's importin-α/β system. In plant cells infected with *Agrobacterium tumefaciens* the effector VirD2 forms a covalently linked complex with the T-DNA in the cytoplasm (Dürrenberger *et al*., 1989). A bipartite NLS at the C-terminus of VirD2 interacts with several Arabidopsis importin-αs and mediates transfer of the T-DNA complex to the nucleus (Ballas and Citovsky, 1997; Bhattacharjee *et al*., 2008). Silencing of *importin-*α*1* or -α*2* in *Nicotiana benthamiana* attenuates nuclear import of several effectors from the oomycete pathogen *Phytophthora infestans* and the *Candidatus Phytoplasma asteris* effector SAP11 (Kanneganti *et al*., 2007; Bai *et al*., 2009). Importin-α-mediated nuclear import is also essential for recognition of the *Xanthomonas campestris* transcription activator-like (TAL) effector AvrBs3 by the pepper *Bs3* gene (Van den Ackerveken *et al*., 1996; Szurek *et al*., 2001). AvrBs3 interacts with plant importin-αs via a C-terminal NLS that is conserved in other TAL effectors (Szurek *et al*., 2001; Schornack *et al*., 2013).

A subcellular localization screen of effector candidates from the Arabidopsis downy mildew pathogen *Hyaloperonospora arabidopsidis* (*Hpa*) revealed that 33% show entirely nuclear localization (Caillaud *et al*., 2012). Despite the prevalence of putative NLSs in effector sequences, a directed Y2H screen of 83 effectors from *Hpa* and *Pseudomonas syringae* detected only two interactions between plant importin-αs and effectors (Mukhtar *et al*., 2011). *Hpa* effector HaRxLL445 interacts with importin-α3/MODIFIER OF SNC1 6 (MOS6) whereas effector HaRxL106 interacts with MOS6, importin-α1, -α2 and -α4. However, results from directed protein–protein interaction assays might not predict with certainty the formation of specific cargo/importin-α complexes in plant cells.

Here we report that *Hpa* effector HaRxL106 binds to the MOS6 armadillo repeat domain via a bipartite NLS with low micro-molar affinity, which is in the range of binding affinities that has been determined for other cargo/importin-α interactions (Marfori *et al*., 2012). We find that small differences in NLS/importin-α binding affinities *in vitro* result in significant changes in cargo/importin-α complex formation in plant cells suggesting that there is significant competition between cargo proteins for binding to importin-αs. A crystal structure of the MOS6 armadillo repeat domain suggests strong conservation of the NLS-binding sites between MOS6 and four other Arabidopsis importin-αs. HaRxL106 binds equally well to these importin-α proteins when they are expressed to comparable levels in *N. benthamiana*. In Arabidopsis leaves, HaRxL106 preferentially forms protein complexes with the most highly expressed importin-α1, -α2 and -α4. This suggests that besides sequence variation in NLS-binding sites, importin-α protein levels can determine which cargo/importin-α complexes form in plant cells.

## Results

### HaRxL106 co-opts the host cell's nuclear import system

**Figure 1:**
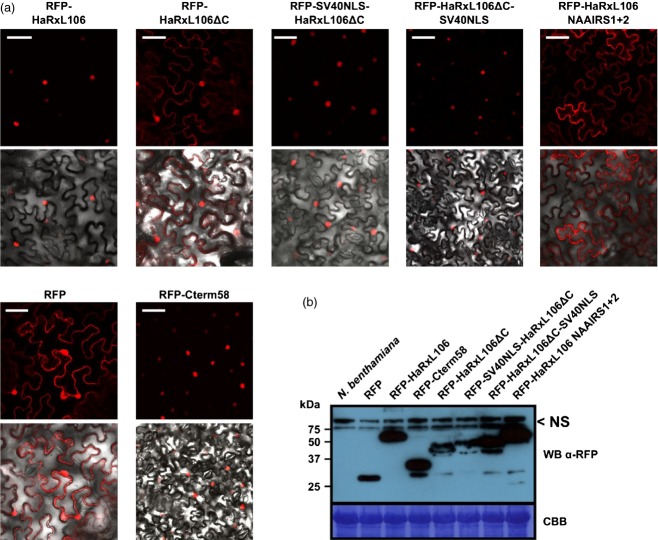
The C-terminal 58 amino acids of HaRxL106 are sufficient and required for active nuclear import.(a) Confocal images of RFP and the indicated RFP–HaRxL106 fusion constructs in epidermal cells of *N. benthamiana*. The images were taken 48 h after infiltration with *A. tumefaciens*. Upper panels show RFP channel, lower panels show RFP channel overlaid on bright field images. Scale bars 50 μm.(b) Western blot of soluble proteins extracts for the RFP fusions used in (a). Samples were harvested 48 h post infiltration with *A. tumefaciens* and probed with α-RFP antibody. NS = non-specific signal of the α-RFP antibody. Coomassie stain shows RubisCO band as loading control.

An RFP-tagged version of HaRxL106, lacking its predicted secretion leader peptide (HaRxL106 amino acids 25–285, referred to as RFP–HaRxL106 from here on), showed entirely nuclear localization when transiently expressed in *N. benthamiana* and when constitutively expressed in Arabidopsis (Figures1a and S1). NLS prediction algorithms identified a putative bipartite NLS at amino acids 239–264 (RGKKRGQTEAPDLEPGLTPKQKRLKR) of HaRxL106 (Kosugi *et al*., 2009; Nguyen Ba *et al*., 2009). By testing a series of N-terminal deletion constructs of HaRxL106 for interaction with MOS6 in a co-immunoprecipitation (co-IP) assay, we confirmed that HaRxL106 amino acids 228–285 (the C-terminal 58 amino acids that encompass the predicted NLS) were sufficient for binding to MOS6 (Figure S2). A construct with a further N-terminal deletion, HaRxL106 amino acids 244–285, did not accumulate to detectable levels preventing us from testing its interaction with MOS6 by co-IP. This construct therefore served as a negative control to exclude non-specific binding of MOS6–GFP to the α-HA affinity resin (Figure S2). Fusion of the 58 C-terminal amino acids of HaRxL106 to RFP (‘RFP–Cterm58’ in Figure1a) shifted the subcellular localization of RFP from nucleo-cytoplasmic to entirely nuclear, demonstrating that this region of HaRxL106 carries a functional NLS. In contrast, deletion of these 58 amino acids (RFP–HaRxL106ΔC) resulted in a nucleo-cytoplasmic distribution that was indistinguishable from RFP alone (Figure1a). Fusion of a heterologous NLS (PKKKRKV) from the SV40 T-antigen to either the N- or C-terminus of the HaRxL106ΔC sequence restored entirely nuclear localization (Figure1a). Despite deletion of the NLS-containing C-terminus, the RFP–HaRxL106ΔC construct still showed residual nuclear localization. This could either be due to a second NLS in the HaRxL106ΔC sequence, or due to elevated passive diffusion of the RFP–HaRxL106ΔC construct (predicted molecular weight 51.3 versus 57.8 kDa for RFP–HaRxL106). To test for presence of an additional NLS we replaced the two clusters of basic amino acids in the predicted bipartite NLS of HaRxL106 by the amino acid sequence NAAIRS, which is unlikely to interfere with protein secondary structure (Wilson *et al*., 1985; Marsilio *et al*., 1991). This RFP–HaRxL106 NAAIRS1+2 fusion protein was more efficiently excluded from nuclei than the RFP–HaRxL106ΔC fusion (Figure1a), suggesting that the residual nuclear localization of the latter construct is due to passive diffusion into nuclei. We confirmed by an α-RFP western blot (Figure1b) that all constructs were expressed and that RFP–HaRxL106 fusions were stable in *N. benthamiana*. Taken together, these data demonstrate that the C-terminal 58 amino acids of HaRxL106 mediate interaction with host importin-αs and that the bipartite NLS is required and sufficient for active nuclear import of the effector.

### HaRxL106 binds to MOS6 directly and with low micro-molar affinity

**Figure 2:**
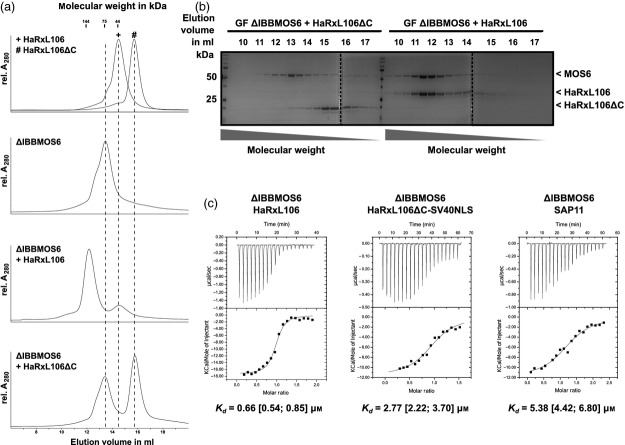
HaRxL106 and MOS6 form a stable complex *in vitro* with a *K*_d_ in the low micro-molar range.(a) Elution volumes of His6-tagged HaRxL106, HaRxL106ΔC and ΔIBBMOS6 on a Superdex HR 200 30/10 size exclusion chromatography column determined by absorption at 280 nm. The upper two panels show elution profiles of the three proteins alone. The lower two panels show elution profiles of mixtures of ΔIBBMOS6 with either HaRxL106 or HaRxL106ΔC at a molar ratio of 1^Δ^^IBBMOS^^6^:2^HaRxL106(ΔC)^.(b) SDS-PAGE of fractions of ΔIBBMOS6/HaRxL106 and the ΔIBBMOS6/HaRxL106ΔC control eluting from the column.(c) ITC binding isotherms and associated fits for the interactions between His6–ΔIBBMOS6 and His6–HaRxL106, His6–HaRxL106ΔC or His6–SAP11. *K*_d_ values are representative of two ITC experiments.

To test for direct interaction between HaRxL106 and importin-α3/MOS6 *in vitro*, we generated *E. coli* expression constructs for the HaRxL106 effector domain (HaRxL106 amino acids 46–285, excluding the N-terminal signal peptide and the RxLR motif), an HaRxL106ΔC version of the same domain (amino acids 46–227) and a truncated version of MOS6 lacking its N-terminal IBB domain. We purified all proteins from the soluble fraction of *E. coli* crude extracts via an N-terminal His6 tag and tested for direct protein–protein interactions by separating protein mixtures on an analytical size exclusion chromatography column (Figure2a,b). When His6-ΔIBBMOS6 was mixed with His6-HaRxL106ΔC, both proteins eluted in separate peaks (Figure2a,b). Instead, when we separated mixtures of His6-ΔIBBMOS6 and His6-HaRxL106, both proteins co-eluted from the column in a complex with a higher molecular weight than the importin-α alone (Figure2a,b). Therefore, the effector domain of HaRxL106 directly binds to the armadillo repeat domain of MOS6 and this interaction requires the HaRxL106 C-terminus encompassing the NLS.

Loss of *importin-*α*3/MOS6* attenuates constitutive immune signalling in the *snc1* mutant background and *mos6* mutants are more susceptible to compatible *Hpa* races and weakly virulent strains of *P. syringae* (Palma *et al*., 2005 and Figure S3). Formally, MOS6 and other importin-αs could therefore also be virulence targets of HaRxL106. However, our finding that HaRxL106 binds to the MOS6 armadillo repeat domain via a typical NLS supports the idea that HaRxL106 binds to importin-αs to co-opt the host cell's nuclear import system. Artificial NLSs with extremely high affinity for importin-α can interfere with cargo release in the nucleus and affect nuclear import (Kosugi *et al*., 2008; Marfori *et al*., 2012). We therefore determined the dissociation constant between ΔIBBMOS6 and the HaRxL106 effector domain by isothermal titration calorimetry (ITC). *In vitro* the two proteins interacted in a 1:1 molar ratio and we determined a *K*_d_ for the ΔIBBMOS6 /HaRxL106 complex in the low micro-molar range (0.54–0.85 μm, Figure2c; for Δ*H* and Δ*S* values see Table S1). To relate this finding to other cargo importin-α interactions, we also determined the dissociation constants of ΔIBBMOS6 complexes with the HaRxL106ΔC–SV40NLS fusion as well as with the Phytoplasma effector SAP11 (Bai *et al*., 2009; Sugio *et al*., 2011). We found that both of these interactions had *K*_d_ values that were only moderately higher than those for the ΔIBBMOS6/HaRxL106 complex (2.22–3.70 μm for HaRxL106ΔC–SV40NLS and 4.42–6.80 μm for SAP11, respectively; Figure2c). Therefore, the HaRxL106 effector domain does not bind to MOS6 with unusually high affinity suggesting that the interaction is a canonical cargo/importin-α interaction.

### A crystal structure of the MOS6 armadillo repeat domain suggests almost identical NLS-binding sites in five Arabidopsis importin-αs

**Figure 3:**
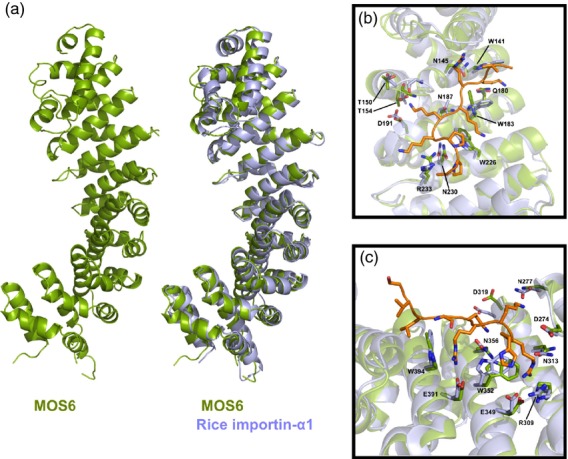
The armadillo repeat domain of MOS6 has the canonical importin-α fold.(a) Crystal structure of the ΔIBBMOS6 protein in cartoon representation and superposition of the armadillo repeat domains of MOS6 (green) and rice importin-α1a (light blue, PDB 4B8O) (Chang *et al*., 2012).(b) Superposition of ΔIBBMOS6 (green) and the ΔIBB variant of rice importin-α1a (light blue, PDB 4B8O) in complex with an SV40NLS (orange) bound at the major NLS-binding site. Residues of rice importin-α1a that contribute to the NLS-binding site and the corresponding MOS6 amino acids are shown in stick representation.(c) Superposition of ΔIBBMOS6 (green) and the ΔIBB variant of rice importin-α1a (light blue, PDB 2YNS) in complex with the B54NLS (orange) bound at the minor NLS-binding site. Residues of rice importin-α1a that contribute to the NLS-binding site and the corresponding MOS6 amino acids are shown in stick representation. Residue labels in (b) and (c) correspond to the MOS6 sequence.

**Figure 4:**
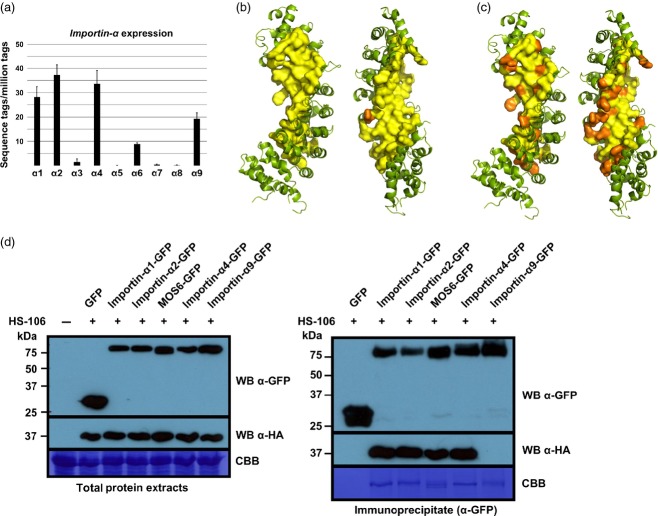
Conservation of the NLS-binding sites of importin-α proteins expressed in Arabidopsis rosette leaves.(a) Sequencing reads of the nine Arabidopsis *importin-*αs detected by RNA-Seq in Col-0 rosette leaves (Asai *et al*., 2014). Error bars show standard deviation (SD) of three biological replicates.(b) Conservation of residues contributing to the MOS6 NLS-binding sites in Arabidopsis importin-α1, -α2, -α4 and -α6. The figure shows the MOS6 armadillo repeat domain and amino acids contributing to the inner concave site of the protein are shown in surface representation. Residues coloured in yellow are conserved in importin-α1, -α2, -α4 and -α6. Orange colour indicates amino acids that diverge from MOS6 in at least one of the other importin-αs. For a sequence alignment of all Arabidopsis importin-α protein sequences, see Wirthmueller *et al*. (2013).(c) Conservation of residues contributing to the MOS6 NLS-binding sites in Arabidopsis importin-α9. Representation as in (b).(d) GFP fusion proteins of importin-α1, -α2, -α4, -α9, MOS6 and free GFP were transiently co-expressed with StrepII-3xHA (HS)-tagged HaRxL106 in *N. benthamiana*. At 48 h post infiltration GFP-tagged importin-αs were IP-ed and co-purifying HS–HaRxL106 was detected by an α-HA western blot. Coomassie stains show RubisCO band in total protein extracts and IP-ed importin-αs in the IP blot. Similar results were obtained in two independent experiments.

We attempted to crystallize ΔIBBMOS6 in complex with either HaRxL106 or an HaRxL106 peptide containing the NLS, but we did not obtain protein crystals of sufficient quality for structure determination. The ΔIBBMOS6 protein on its own formed diffracting protein crystals and enabled us to determine the crystal structure of the ΔIBBMOS6 protein at 2.9 Å resolution (Figure3a and Table S2; Data S4; PDB identifier 4TNM). Like other importin-α proteins from yeast, mammals and rice, ΔIBBMOS6 forms 10 armadillo repeats with strong conservation of residues that contribute to the major and minor NLS-binding sites (Marfori *et al*., 2011). We superposed the ΔIBBMOS6 structure onto the structure of rice importin-α1a in complex with a SV40NLS (Chang *et al*., 2012). This revealed that essentially all amino acids of rice importin-α1a, that make direct contact to the SV40NLS at the major and minor NLS-binding sites, are conserved in MOS6 (Figure3b,c). The Arabidopsis genome encodes nine importin-αs (Merkle, 2011; Wirthmueller *et al*., 2013). Despite a high level of sequence conservation in the H3 helices that form the NLS-binding sites, knock-out of a single *importin-*α gene can lead to mutant phenotypes (Palma *et al*., 2005; Bhattacharjee *et al*., 2008). One possible determinant of specificity is variation in the importin-α NLS-binding sites that would lead to specific interaction with distinct NLSs. We determined the conservation of the NLS-binding sites of importin-αs expressed in rosette leaves by homology modelling based on the ΔIBBMOS6 structure. In RNA-sequencing experiments (Asai *et al*., 2014) we reliably detected sequencing reads of six *importin-*α genes in rosette leaf tissue (*importin-*α*1*, -α*2*, -α*3/MOS6*, -α*4*, -α*6* and -α*9*). Out of these, *importin-*α*1*,*-*α*2* and *-*α*4* had the highest expression levels, followed by *importin-*α*9*, -α*6* and -α*3/MOS6* (Figure4a). We found that residues contributing to the MOS6 NLS-binding site are strongly conserved in importin-α1, -α2, -α4 and -α6 (Figure4b) whilst these residues are less conserved in importin-α9 (Figure4c). Consistent with a conserved NLS-binding site, StrepII-3xHA (HS)-tagged HaRxL106 bound equally well to GFP-tagged importin-α1, -α2, -α4 and MOS6 in co-IPs (Figure4d). In contrast, HaRxL106 did not co-IP with importin-α9 (Figure4d). We further tested which importin-αs co-purify with HaRxL106 in Arabidopsis. We IP-ed an YFP–HaRxL106 fusion protein from a stable transgenic line (see Data S4) and identified co-purifying importin-α proteins by liquid chromatography coupled with mass spectrometry (LC-MS/MS). In three independent replicates we consistently detected unique peptides from importin-α1, -α2 and -α4 in IPs of YFP–HaRxL106, whilst we found only a single importin-α peptide in one out of three control IPs from wild-type plants or a line expressing GFP (Table1 and Data S1 and S2). Thus, in Arabidopsis rosette leaves, HaRxL106 appears to bind preferentially to the three importin-αs with the highest mRNA expression levels.

**Table 1:** Number of unique importin-α tryptic peptides identified by LC-MS/MS following immunoprecipitation of YFP–HaRxL106 from Arabidopsis

	Experiment 1		Experiment 2		Experiment 3	
	GFP	YFP–HaRxL106	GFP	YFP–HaRxL106	Col-0	YFP–HaRxL106
Importin-α1	–	4	–	2	–	4
Importin-α2	–	24	1	10	–	13
Importin-α3	–	5	–	4	–	4

### Small differences in NLS-cargo/importin-α affinities *in vitro* significantly affect formation of transport complexes in plant cells

**Figure 5:**
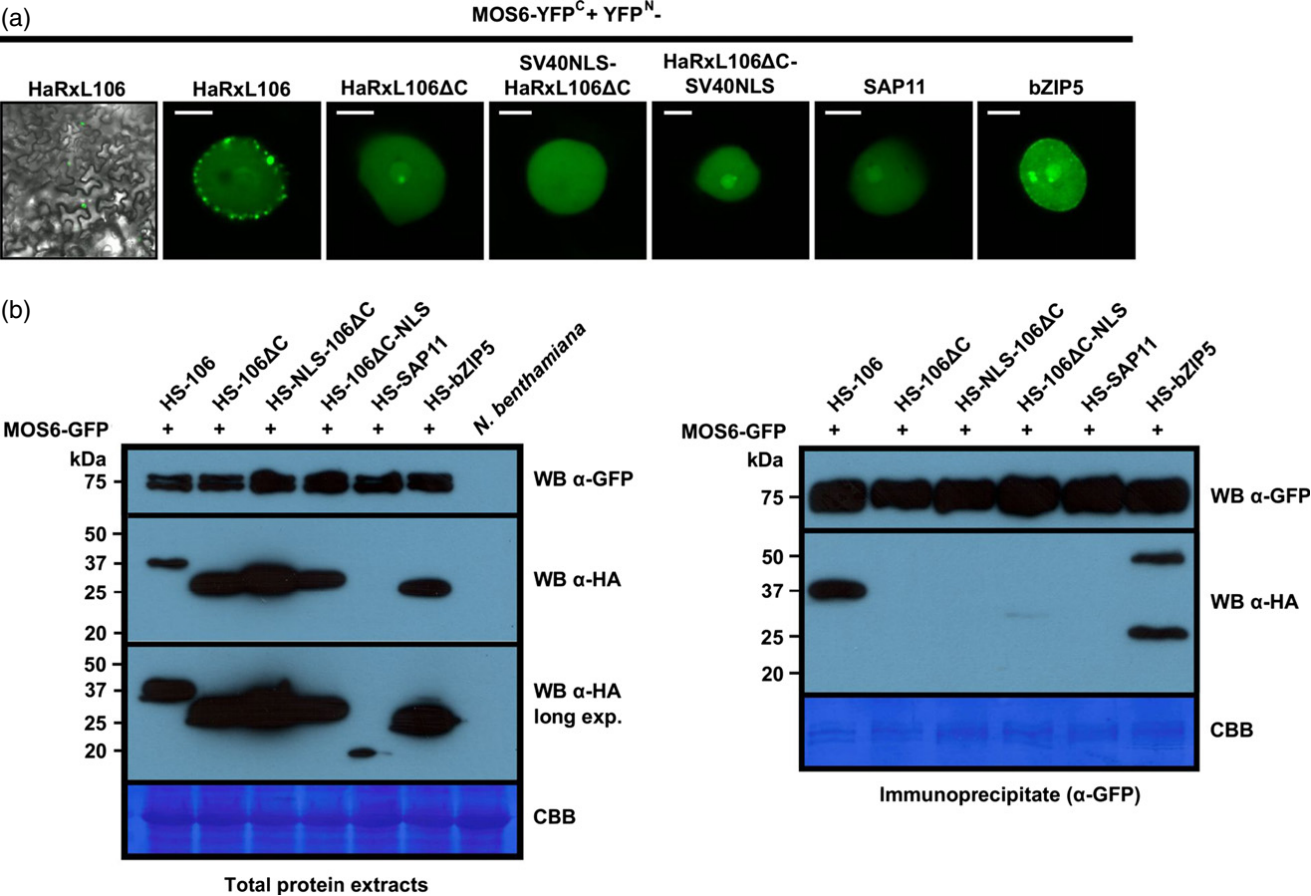
The HaRxL106 NLS mediates stronger complex formation with importin-αs than the SV40NLS in plant cells.(a) BiFC between MOS6–YFP^C^ and the indicated YFP^N^-tagged NLS-cargo proteins in nuclei of *N. benthamiana* 48 h post infiltration. Images are representative of at least 10 nuclei analysed. Scale bars 5 μm.(b) MOS6–GFP was transiently co-expressed with the indicated StrepII-3xHA (HS)-tagged NLS-cargo proteins in *N. benthamiana*. At 48 h post infiltration MOS6–GFP was IP-ed and co-purifying HS-tagged proteins were detected by an α-HA western blot. Coomassie stains show RubisCO band in total protein extracts and IP-ed importin-αs in the IP blot. Similar results were obtained in two independent experiments.

A previous study reported that although a double Lys to Ala mutation in the NLS of the yeast ribosomal protein Rpl25p resulted only in an approximately threefold reduced binding affinity to its cognate import receptor Kap123p/importin-β4, this mutation significantly reduced nuclear import rates in yeast (Timney *et al*., 2006). The authors explained this discrepancy by non-specific competition for importin-β binding by other cytoplasmic proteins as it could be mimicked by an *E. coli* protein extract (Timney *et al*., 2006). As the HaRxL106/ΔIBBMOS6 complex has an approximately 4–8-fold lower *K*_d_ when compared with ΔIBBMOS6 complexes with HaRxL106ΔC–SV40NLS or SAP11, we tested if this difference in *K*_d_ affects formation of MOS6/cargo complexes in *N. benthamiana* cells. To this end, we generated a MOS6–YFP^C^ bimolecular fluorescence complementation (BiFC) expression construct and co-expressed this fusion protein with YFP^N^-tagged cargo proteins in epidermal cells of *N. benthamiana*. Apart from an YFP signal in the nucleoplasm, which we observed for all BiFC pairs tested and therefore might result from spontaneous association of the YFP N- and C-terminal halves, we found that co-expression of YFP^N^–HaRxL106 with MOS6–YFP^C^ resulted in speckles at the nuclear rim (Figure5a). Speckle formation was dependent on the HaRxL106 C-terminus as we did not observe them with the YFP^N^–HaRxL106ΔC construct. Although the SV40NLS is sufficient to restore entirely nuclear localization of HaRxL106ΔC (Figure1a), fusion of the SV40NLS to either the HaRxL106ΔC N- or C-terminus did not result in speckles at the nuclear periphery in BiFC (Figure5a). Similarly, we did not observe speckles in BiFC experiments between YFP^N^–SAP11 and MOS6–YFP^C^. Although the molecular basis of speckle formation in this over-expression system remains unknown, we suggest that they may represent MOS6/HaRxL106 complexes that cannot be disassembled as efficiently as other importin-α/cargo complexes on the nucleoplasmic side of the NPC.

To exclude the possibility that the YFP speckles of the YFP^N^–HaRxL106/MOS6–YFP^C^ interaction are simply due to differences in protein levels compared with other YFP^N^-tagged cargos, we performed co-IPs between transiently expressed MOS6–GFP and HS-tagged cargo proteins in *N. benthamiana* cell extracts. IP of MOS6–GFP co-purified HS-HaRxL106, but not the corresponding HaRxL106ΔC construct (Figure5b). Although the SV40NLS was sufficient to restore nuclear import of the RFP–HaRxL106ΔC protein (Figure1a), we detected no or only very weak interactions between MOS6–GFP and HaRxL106ΔC constructs that carry the SV40NLS either at the N- or C-terminus (Figure5b). HS–SAP11 accumulated to lower levels than all other cargo proteins in the total extract and we did not detect SAP11 binding to MOS6 in co-IPs (Figure5b). The BiFC and co-IP data demonstrate that in plant cells the NLS of HaRxL106 forms more stable complexes with MOS6 than those mediated by the SAP11 or SV40NLS. We next addressed if this property is unique to HaRxL106. A BLAST search with the NLS of HaRxL106 against the TAIR Arabidopsis protein database (v. 10) identified a Lys-rich sequence from the transcription factor bZIP5 (AT3G49760) as close match (Figure S4). In co-IPs, we detected a strong interaction between MOS6–GFP and HS–bZIP5 suggesting that formation of these stable complexes with MOS6 is not a unique feature of the HaRxL106 NLS (Figure5b). In addition to the HS–bZIP5 monomer (approximately 25 kDa), we also detected a approximately 50 kDa band in IPs that might correspond to a bZIP5 dimer (Figure5b). In BiFC experiments the YFP^N^–bZIP5/MOS6–YFP^C^ combination formed speckles at the nuclear periphery although they were less intense compared to those observed with YFP^N^–HaRxL106 (Figure5a). Taken together, the BiFC and co-IP results show that, despite the similar *K*_d_ values we determined for select NLS-cargo/MOS6 complexes *in vitro* (Figure2c), there are strong differences in transport complex formation in plant cells (Figure5).

### NLS-cargos compete with other proteins for binding to importin-αs in plant cells

**Figure 6:**
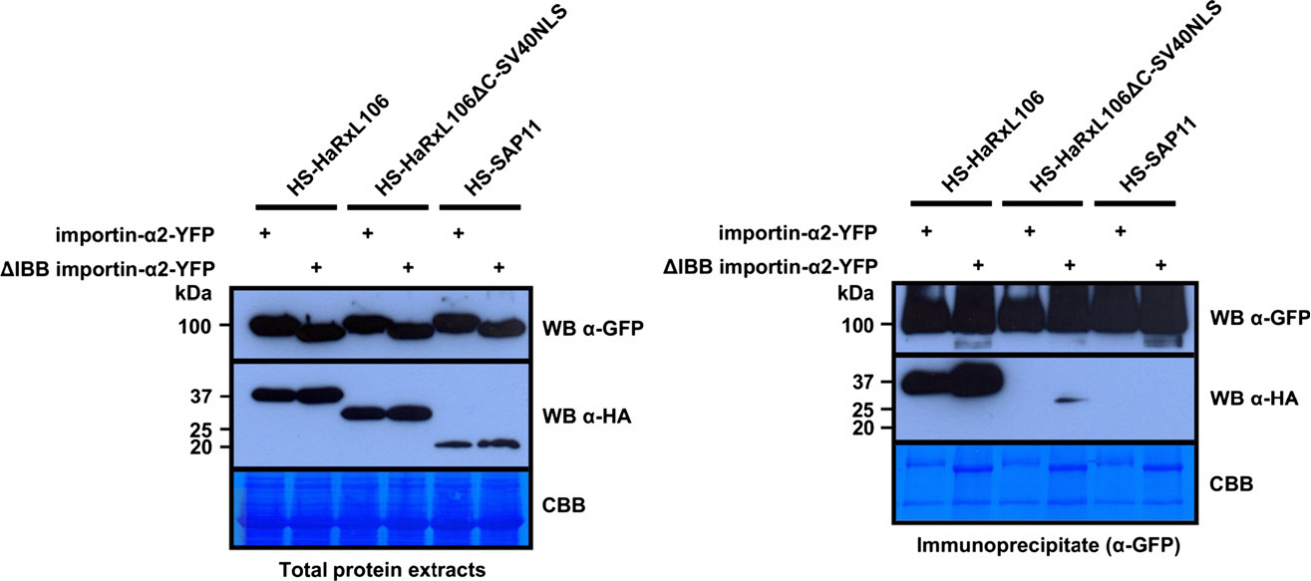
Competition for importin-α binding in plant cells is not only mediated by the IBB domain.Importin-α2–YFP or the corresponding ΔIBB constructs were transiently co-expressed with the indicated HS-tagged NLS-cargo proteins in *N. benthamiana*. At 48 h post infiltration YFP-tagged importin-αs were IP-ed and co-purifying StrepII-3xHA (HS)-tagged proteins were detected by an α-HA western blot. Coomassie stains show RubisCO band in total protein extracts and IP-ed importin-αs in the IP blot. Similar results were obtained in two independent experiments.

Our finding that small differences in *K*_d_ values determined *in vitro* translate into substantial differences in NLS/importin-α complex formation in plant cells could be due to competition by other cytoplasmic proteins for importin-α binding. As we used the non-auto-inhibited ΔIBB variant of MOS6 to determine *K*_d_ values *in vitro* (Figure2c), competition for MOS6 binding in plant cells could be either due to the auto-inhibiting function of the IBB domain or due to the presence of other competing proteins in the cytoplasm. As importin-αs are over-expressed in the transient expression system, negation of IBB auto-inhibition by endogenous importin-βs is likely to be negligible (Cardarelli *et al*., 2009). To distinguish between competition by the IBB domain and other cytoplasmic proteins we used the *N. benthamiana* transient expression system to test if HaRxL106, HaRxL106ΔC–SV40NLS and SAP11 differ in their abilities to form complexes with importin-α2 and ΔIBB importin-α2 (importin-α2 and MOS6 show comparable binding to HaRxL106, Figure4d). The ΔIBB variant of importin-α2–YFP protein co-purified slightly more HS–HaRxL106 than the full-length importin-α2. This could either be due to the lack of auto-inhibition by the IBB domain or due to the higher protein levels of the ΔIBB importin-α2–YFP construct when compared with importin-α2–YFP (see CBB stain in Figure6). However, ΔIBB importin-α2–YFP still co-purified HS–HaRxL106 more efficiently than HaRxL106ΔC–SV40NLS or SAP11 (Figure6). Therefore, the differential complex formation in plant cells is not only a result of enhanced auto-inhibition by the IBB domain of over-expressed importin-αs but is due to additional competing factors in plant cell extracts. These findings suggest that endogenous NLS-cargos in plant cells compete with other proteins for binding to importin-α receptors and that NLS-cargo concentration and affinity for importin-αs determine formation of ternary transport complexes in the cytosol.

## Discussion

### Co-option of the importin-α/β nuclear transport pathway by HaRxL106 and other pathogen effectors

The contribution of importin-α3/MOS6 to plant immunity makes it a putative virulence target of pathogen effectors. Here we provide several lines of evidence suggesting that HaRxL106 binds to MOS6 and other importin-αs as a cargo protein but does not interfere with their function as NTRs:

- HaRxL106 binds MOS6 exclusively via a peptide that fits to the consensus sequence of bipartite NLSs (Figure1; Marfori *et al*., 2012)
- *In vitro*, the *K*_d_ of the HaRxL106/MOS6 complex is only slightly lower than that mediated by the canonical SV40NLS (Figure2c). In contrast synthetic NLSs, that interfere with nuclear transport, bind importin-αs with an affinity that is approximately one order of magnitude higher than that of the SV40NLS (Kosugi *et al*., 2008).
- *In vivo* the Arabidopsis transcription factor bZIP5 and HaRxL106 bind to MOS6 with similar efficiency (Figure5).
- We have not observed that over-expression of HaRxL106 in *N. benthamiana* or *A. thaliana* leads to cell death, as one might expect if HaRxL106 were a strong inhibitor of nucleo-cytoplasmic transport.

Based on the NLS peptide-mediated mode of binding to importin-αs and the *K*_d_ of the HaRxL106/MOS6 interaction we conclude that HaRxL106 is a cargo protein of Arabidopsis importin-αs. Notably, the molecular weight of several effectors that exploit the plant's nuclear transport system is below the molecular weight exclusion limit of NPCs (Wang and Brattain, 2007). Like HaRxL106 (27 kDa), *P. infestans* NUK7 (47 kDa) and SAP11 (11 kDa) co-opt the importin-α/β pathway for efficient nuclear import (Howard *et al*., 1992; Shurvinton *et al*., 1992; Kanneganti *et al*., 2007; Bai *et al*., 2009). Therefore, even without an NLS, these effector proteins would be expected to enter the host cell nucleus by passive diffusion. Considering that effector protein levels might be relatively low in an infected cell, evolution of NLS sequences in these proteins may represent a mechanism for enhanced transport to ensure efficient delivery to the nucleus when compared with passive diffusion.

### Functional affinity limits of NLS/importin-α interactions

Dissociation constants for several NLS/importin-α complexes from yeast, mammals and plants have been determined (Hübner *et al*., 1999; Hodel *et al*., 2001, 2006; Timney *et al*., 2006; Kosugi *et al*., 2008; Chang *et al*., 2012). Based on these results it has been suggested that *K*_d_ values for canonical NLS-binding to importin-αs are in the range of approximately 10 nm to 1 μm (Marfori *et al*., 2012). The *K*_d_ values we determined for HaRxL106, HaRxL106ΔC–SV40NLS and SAP11 binding to the non-auto-inhibited MOS6 protein are at or beyond the upper limit of this interval and we would expect even higher *K*_d_ values for complexes formed between full-length MOS6 and these cargo proteins. One explanation for this discrepancy may be the experimental method used to determine *K*_d_ values. The 10 nm to 1 μm interval is mainly based on assays that require binding of one protein to a surface, such as plate binding assays (Hübner *et al*., 1999; Timney *et al*., 2006; Chang *et al*., 2012) or surface plasmon resonance (Kosugi *et al*., 2008). In contrast, we determined the *K*_d_ values reported here by ITC. Two other reports have used ITC to determine dissociation constants for NLS/importin-α complexes. Ge *et al*. (2011) measured a *K*_d_ of 3.03 ± 0.95 μm for binding of the NLS peptide from the rat transcription factor ChREBP to importin-α. Lott *et al*. (2011) obtained a *K*_d_ of 48.7 ± 6.5 μm for binding of the NLS peptide from human phospholipid scramblase 4 to the non-auto-inhibited form of mouse importin-α2. Thus, it appears that *K*_d_ values in the low micro-molar range are not unusual when determined by ITC and that differences to previously reported functional *K*_d_ values in the low nano-molar range are probably due to different methods applied.

### Cargo proteins compete for binding to importin-α receptors in plant cells

Although the NLSs from HaRxL106, SAP11 and the SV40NLS bind to the non-auto-inhibited form of MOS6 with comparable affinities *in vitro*, we observed substantial differences in cargo/importin-α complex formation in plant cells (BiFC, Figure5a) and plant cell extracts (co-IP, Figure5b). A 4–8-fold difference in *K*_d_ values is unlikely to cause significant differences in complex formation unless there is competition for binding to the receptor. As the ΔIBB variant of importin-α2 co-purifies HaRxL106 much more efficiently than HaRxL106ΔC–SV40NLS and SAP11, this competitive effect is not mediated by the IBB domain (Figure6). Our results are consistent with nuclear import experiments in yeast and in mammalian cells demonstrating that a 2–7-fold difference in *K*_d_ values alters nuclear import kinetics (Efthymiadis *et al*., 1997; Xiao *et al*., 1998; Hodel *et al*., 2006; Timney *et al*., 2006). Timney *et al*. (2006) proposed that other cytoplasmic proteins non-specifically compete with binding of ribosomal cargo proteins to importin-β NTRs, thus explaining the discrepancy between *in vitro* and *in vivo* experiments. The same macromolecular crowding effect could also explain the difference between cargo/importin-α complex formation *in vitro* and in plant cells. However, we would expect that over-expression of cargos and importin-αs combined with several-fold dilution of other potentially competing proteins in a plant cell extract [typical protein concentration 6.5 mg ml^−1^ versus estimated protein concentration in the cytosol 100–200 mg ml^−1^ (Ellis, 2001; Zeskind *et al*., 2007)] diminishes macromolecular crowding. It is therefore surprising that we still observed differences in cargo/importin-α complex formation in co-IPs. It is conceivable that in addition to non-specific competition by bulk cellular proteins other NLS-cargos compete with binding to importin-αs and that competition is stronger in the approximately 4–8-fold higher *K*_d_ range of the SV40 and SAP11 NLSs when compared to the NLS of HaRxL106.

### Conservation of the NLS-binding site in plant importin-αs

The nine Arabidopsis importin-α proteins show approximately 26% overall sequence identity. However, when only the H3 helices of ARM repeats 1–8 that contribute the NLS-binding sites are considered, the sequence identity is approximately 45% (Wirthmueller *et al*., 2013). This conservation of the H3 helices allowed us to build homology models for the armadillo repeat domains of other Arabidopsis importin-αs based on the ΔIBBMOS6 structure. Superposition of individual models with the ΔIBBMOS6 structure revealed an almost complete conservation of the major and minor NLS-binding sites in five out of six importin-αs expressed in rosette leaves (importin-α1, -α2, -α3, -α4 and -α6) (Figure4b,c). Our observation that HaRxL106 binds equally well to importin-α1, -α2, -α4 and MOS6 in plant cell extracts (Figure4d) is in agreement with a conserved NLS-binding site on these importin-αs. Given this redundancy, it is interesting that genetic knock-out of a single *importin-*α gene can lead to mutant phenotypes (Palma *et al*., 2005; Bhattacharjee *et al*., 2008). We found that YFP–HaRxL106, IP-ed from transgenic Arabidopsis lines, predominantly interacts with importin-α1, -α2 and -α4, which have the highest expression levels in rosette leaves (Table1 and Figure4a). Tissue-specific differences in *importin-*α expression levels might therefore determine each importin-αs contribution to nuclear transport in the particular cell type. Bhattacharjee *et al*. (2008) reported that knock-out of *importin-*α*4*, but not -α*1*, -α*2* or *MOS6*, leads to lower *A. tumefaciens* transformation rates in Arabidopsis root tissue. Based on available mRNA expression data (Hruz *et al*., 2008; Wirthmueller *et al*., 2013), *importin-*α*4* has the highest expression level in root cells. Bhattacharjee *et al*. (2008) also found that several *importin-*α paralogs can complement the reduced transformation rates of the *importin-*α*4* mutant when expressed under control of the tissue non-specific 35S promoter, supporting the hypothesis that tissue-specific expression levels of single *importin-*α genes might determine their contribution to nuclear transport. The NLS of yeast ribosomal protein Rpl25 has comparable affinities for the importin-βs Kap123p and Kap121p. However, due to higher cellular levels of Kap123p, this importin-β acts as the primary transport receptor in yeast (Timney *et al*., 2006). Our results suggest that: (i) protein levels of plant importin-αs; and (ii) the affinity of an NLS for a particular importin-α are two major factors that determine which NLS-cargo/importin-α complexes form in the plant cell cytoplasm. However, other possible sources of specificity such as different preferences for association of importin-αs with importin-βs or post-translational modification of importin-α/β and NLS flanking sequences have not thoroughly been addressed in plants and might add a further layer of regulation to nuclear import.

## Experimental Procedures

### Plants and growth conditions

Growth conditions for *N. benthamiana* and Arabidopsis have been described (Fabro *et al*., 2011; Segonzac *et al*., 2011). The *mos6-1* and *mos6-2* mutants have been described (Palma *et al*., 2005). The *mos6-4* T-DNA insertion line (SALK 025919) was obtained from NASC. Transgenic Arabidopsis plants expressing YFP– and RFP–HaRxL106 were generated by transforming ecotype Col-0 with *A. tumefaciens* strain GV3101 pMP90^RK^ carrying pENS-YFP–HaRxL106 and *A. tumefaciens* strain GV3101 pMP90 carrying pH7WGR2–HaRxL106, respectively (Logemann *et al*., 2006).

### Pathogen assays

For bacterial growth assays 4-week-old plants were vacuum-infiltrated with bacterial suspensions of 1 × 10^5^ cfu ml^−1^ in 5 mm MgCl_2_ and 0.0015% Silwett L-77 of *P. syringae* DC3000 ΔAvrPto/AvrPtoB (Lin and Martin, 2005) or ΔCEL (Alfano *et al*., 2000) and bacterial titres were determined at the day of infiltration and 3 days post inoculation by plating dilution series of extracts from infected leaves on selective media.

### Transient expression

*A. tumefaciens* GV3101 and GV3103 bacteria were grown on selective plates, resuspended in 10 mm MgCl_2_ 10 mm MES pH 5.6 and incubated with 100 mm acetosyringone for 2 h at RT. Each strain was mixed with *A. tumefaciens* strain GV3101 expressing the silencing suppressor 19 K at a ratio of 1:3^[19K]^. For co-expression the strains were mixed in a 1:1:3^[19K]^ ratio. Leaves of 3–4-week-old *N. benthamiana* plants were infiltrated with a syringe and leaves were harvested or imaged 48–72 h later.

### Protein extraction from *N. benthamiana*, co-IP and western blot

Protein extracts were prepared by grinding *N. benthamiana* or Arabidopsis leaf material in liquid nitrogen to a fine powder followed by resuspension in extraction buffer [50 mm Tris, 150 mm NaCl, 10% glycerol, 1 mm EDTA, 5 mm DTT, 1× protease inhibitor cocktail (Sigma, http://www.sigmaaldrich.com), pH 7.5] at a ratio of 2 ml buffer per 1 g leaf material. The extracts were centrifuged at 17 000 ***g*** 4°C 20 min and the supernatant was either boiled in sodium dodecyl sulphate (SDS) sample buffer for western blots or used for co-IPs. For western blots protein samples were separated by SDS-PAGE and electro-blotted onto polyvinylidene difluoride membrane. Antibodies used were α-HA 3F10 (Roche, http://www.roche.com), α-GFP 210-PS-1GP (Amsbio, http://www.amsbio.com), α-RFP-biotin ab34771 (Abcam, http://www.abcam.com). For co-IPs a fraction of the supernatant was saved as ‘input’ sample and 20 μl GFP-beads (GFP-Trap_A; Chromotek, http://www.chromotek.com) or HA-beads (Sigma) were added to 1.4 ml of the remaining supernatant. The samples were incubated on a rotating wheel at 4°C for 2 h followed by collecting the beads by centrifugation at 1200 ***g*** and 4°C for 1.5 min. The beads were washed 3–4 times with 1 ml extraction buffer and then boiled in SDS sample buffer to elute protein from the beads.

### Isothermal titration calorimetry

ITC experiments were performed using a MicroCal 205 calorimeter (Malvern, http://www.malvern.com) in high gain mode at 25°C with all proteins diluted in buffer 20 mm HEPES, 150 mm NaCl, pH 7.5. His6–ΔIBB–MOS6 protein was pipetted into the sample chamber at 43–54 μm concentration and was titrated with His6-tagged HaRxL106, HaRxL106ΔC or SAP11 at concentrations between 320 and 940 μm. Two microlitre injections with 120 sec pause intervals were performed up to a cumulative volume of 38 μl. Binding isotherms were fitted to the integrated calorimetric data using Origin software (OriginLab, http://www.originlab.com). Control reactions titrating buffer into ΔIBBMOS6 showed that the heat of dilution was <0.1 Kcal mol^−1^ of injectant and therefore comparable with the values obtained at the end point of each titration. At least one technical replicate for each ITC experiment was performed and gave similar results.

### Confocal microscopy

*N. benthamiana* or Arabidopsis leaf discs were mounted onto microscopy slides in 60% glycerol or water and analysed on a Leica DM6000B/TCS SP5 confocal microscope (Leica Microsystems, http://www.leica-microsystems.com) with the following excitation wavelengths: YFP, 516 nm; RFP, 561 nm.

### Analytical size exclusion chromatography

Analytical size exclusion chromatography was performed using a Superdex 200 HR 10/30 column (GE Healthcare, http://www.gelifesciences.com) in 50 mm HEPES, 150 mm NaCl, pH 7.0. His6–ΔIBB–MOS6 protein was diluted to a concentration of 2 mg ml^−1^ and incubated with a 1 m excess of either His6–HaRxL106 or His6–HaRxL106ΔC for 1 h at 4°C. The samples were centrifuged at 17 000 ***g*** 4°C 20 min and 0.5 ml of the cleared supernatant was loaded on the column. The column was eluted at a flow rate of 0.5 ml min^−1^ with two column volumes of buffer and 0.5 ml fractions were analysed by SDS-PAGE.

### Plasmids and oligo-nucleotides

For a list of oligo-nucleotides and plasmids used in this study see Data S3 and S4.

## Supporting Information

**Figure S1.**RFP-HaRxL106 is entirely nuclear localized when expressed as transgene in *Arabidopsis thaliana*.

**Figure S2.**HaRxL106 amino acids 228–285 are sufficient for MOS6 binding.

**Figure S3.***mos6* mutants are more susceptible to *P. syringae* strains with reduced effector repertoires.

**Figure S4.** Sequence alignment between HaRxL106 amino acids 232–279 and Arabidopsis bZIP5 amino acids 61–120.

**Table S1.** Stoichiometry, Δ*H* and Δ*S* values for ITC experiments shown in Figure 2(c).

**Table S2.** X-ray data collection, refinement, and validation statistics.

**Data S1.** Proteins identified by LC-MS/MS in immuno-precipitates of YFP-HaRxL106 transgenics and controls.

**Data S2.** Maxima of best Mascot ion scores and total spectrum counts for peptides identified by LC-MS/MS.

**Data S3.** List of oligo-nucleotides and pENTR plasmids used in this study.

**Data S4.** Experimental procedures.
