# Supplementary figures and images for: Probing formation of cargo/importin-α transport complexes in plant cells using a pathogen effector
Source: Plant J. 2014 Nov 17;81(1):40–52. doi: 10.1111/tpj.12691 (PMC4350430; doi:10.1111/tpj.12691)

**35S-RFP**

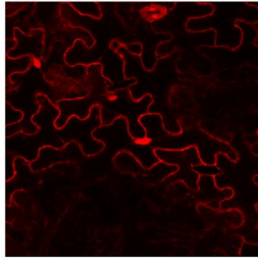

**35S-RFP-HaRxL106**

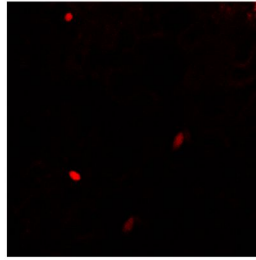

**RFP channel**

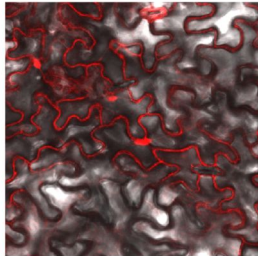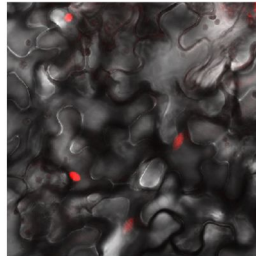

**overlay with  
BF image**

Supplement: Supplementary file 1 — Figure S1.RFP-HaRxL106 is entirely nuclear localized when expressed as transgene in Arabidopsis thaliana. [file tpj0081-0040-sd1.pdf]

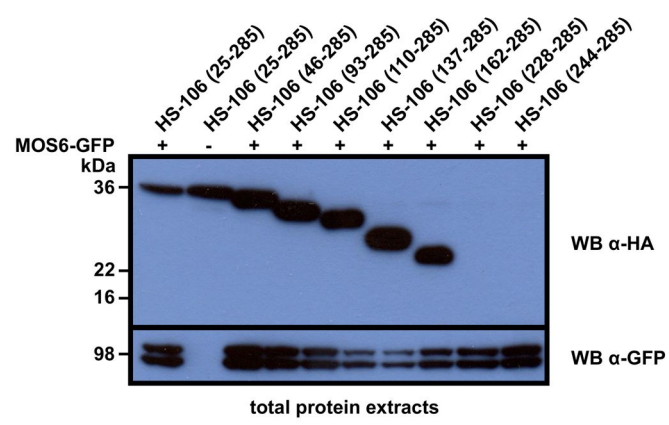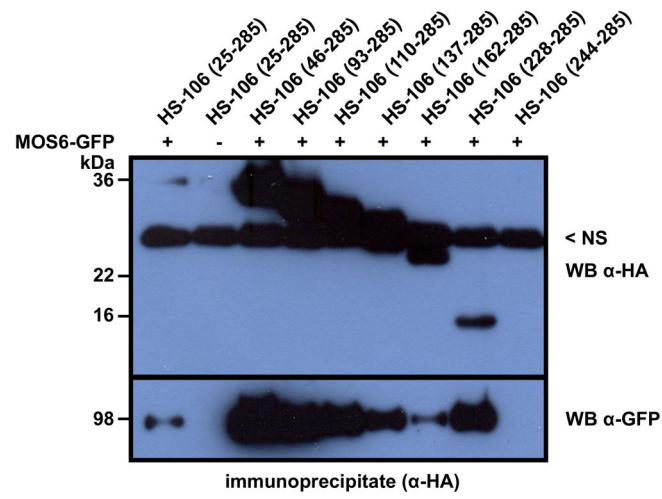

Supplement: Supplementary file 2 — Figure S2.HaRxL106 amino acids 228–285 are sufficient for MOS6 binding. [file tpj0081-0040-sd2.pdf]

(a)

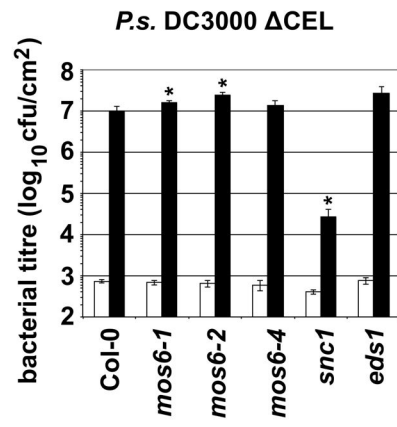

(b)

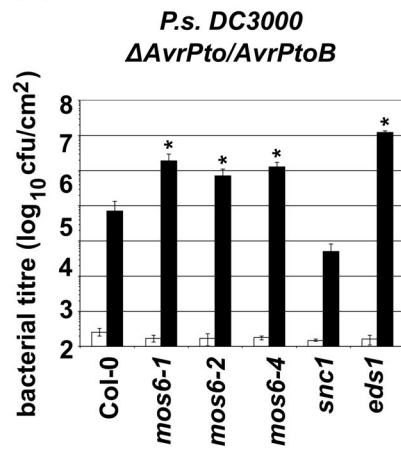

Supplement: Supplementary file 3 — Figure S3.mos6 mutants are more susceptible to P. syringae strains with reduced effector repertoires. [file tpj0081-0040-sd3.pdf]
