# Supplementary material for: Probing formation of cargo/importin-α transport complexes in plant cells using a pathogen effector
Source: Plant J. 2014 Nov 17;81(1):40–52. doi: 10.1111/tpj.12691 (PMC4350430; doi:10.1111/tpj.12691)
Supplement: Supplementary file 4 — Figure S4. Sequence alignment between HaRxL106 amino acids 232–279 and Arabidopsis bZIP5 amino acids 61–120. [file tpj0081-0040-sd4.pdf]

```
106NLS      -----VEGTESRGKKRGQTEAPDLEPGLTPKQKRLKRMELQRVKKILLNINL--------
bZIP5       SDMSPTDNTDERKKKRKLSNRESAKRSREKKQKHLEEMSIQLNQLKIQNQELKNQLRYVL
                 .: *:.* ***  ::  . : .   ***:*:.*.:*  :  : * :*
```
