# Supplementary material for: Probing formation of cargo/importin-α transport complexes in plant cells using a pathogen effector
Source: Plant J. 2014 Nov 17;81(1):40–52. doi: 10.1111/tpj.12691 (PMC4350430; doi:10.1111/tpj.12691)
Supplement: Supplementary file 5 — Table S1. Stoichiometry, ΔH and ΔS values for ITC experiments shown in Figure 2(c). [file tpj0081-0040-sd5.docx]

**Table S1.** Stochiometry, ΔH and ΔS values for ITC experiments shown in Figure 2c.

|  | HaRxL106 | HaRxL106ΔC-  SV40NLS | SAP11 |
| --- | --- | --- | --- |
| number of binding sites | 0.950 ±0.0127 | 0.981 ±0.0271 | 1.28 ±0.0381 |
| ΔH cal/mol | -1.734 x 10^4^ ±357.5 | -1.162 x 10^4^ ±567.5 | -1.183 x 10^4^ ±516.4 |
| ΔS cal/mol/deg | -29.9 | -13.6 | -15.6 |
