# Supplementary material for: Probing formation of cargo/importin-α transport complexes in plant cells using a pathogen effector
Source: Plant J. 2014 Nov 17;81(1):40–52. doi: 10.1111/tpj.12691 (PMC4350430; doi:10.1111/tpj.12691)
Supplement: Supplementary file 6 — Table S2. X-ray data collection, refinement, and validation statistics. [file tpj0081-0040-sd6.docx]

**Table S2.** X-ray data collection, refinement, and validation statistics

| Data collection |  |
| --- | --- |
| Beamline | Diamond Light Source-I02 |
| Wavelength (Å) | 0.976 |
| Space group | H3 |
| Unit cell parameters  a, b, c (Å) | 127.5, 127.5 89.78 |
| Unique reflections^*^ | 11858 (1933) |
| Resolution (Å)^*^ | 63.76 – 2.90 (3.08 – 2.90) |
| R_merge_ (%)^*, #^ | 0.047 (0.297) |
| I/σ(I)^*, #^ | 8.8 (2.6) |
| Completeness (%)^*, #^ | 98.3 (98.8) |
| Multiplicity^*, #^ | 2.7 (2.7) |
| Refinement |  |
| Resolution | 69.66 – 2.90 (2.98 – 2.90) |
| R_work_ (%)^*, &^ | 0.2207 (0.3350) |
| R_free_ (%)^*, &^ | 0.2626 (0.3520) |
| Number of atoms | 3138 |
| B-factors^&^ | 106.386 |
| rmsd |  |
| Bond length (Å)^&^ | 0.004 |
| Bond angles (º)^&^ | 0.780 |
| Ramachandran favored (%)^+^ | 96.7 |
| Ramachandran outliers (%)^+^ | 0.5 |

^*^Values in parenthesis correspond to the highest resolution bin.

^#^Values reported by AIMLESS (Evans and Murshudov, 2013).

^&^Values reported by REFMAC (v5.8.0071) (Murshudov *et al.*, 2011).

^+^Values reported by MOLPROBITY (Chen *et al.*, 2010).
