# Supplementary material for: Probing formation of cargo/importin-α transport complexes in plant cells using a pathogen effector
Source: Plant J. 2014 Nov 17;81(1):40–52. doi: 10.1111/tpj.12691 (PMC4350430; doi:10.1111/tpj.12691)
Supplement: Supplementary file 10 — Data S4. Experimental procedures. [file tpj0081-0040-sd10.docx]

**Supporting experimental procedures**

***Plasmids and oligo-nucleotides***

For a list of oligo-nucleotides and pENTR/D-Topo vectors used in this study see supporting data S3. Binary plasmids for transient and stable plant transformation were generated by Gateway LR reactions between these pENTR/D-Topo clones and Gateway destination vectors. To generate pENS-YFP-HaRxL106 pENTR/D-Topo clone 1 was recombined with pENS-YFP (Jane Parker, MPIPZ, Cologne). To generate pH7WGR2-HaRxL106, N- and C-terminal deletion variants and fusions with the SV40NLS pENTR/D-Topo clones 1-11 were recombined into pH7WGR2 (Karimi *et al.*, 2002). To generate importin-α-GFP fusion constructs pENTR/D-Topo clones 12-16 were recombined into pK7WGF2 (Karimi *et al.*, 2002). The MOS6-GFP pBS-GFP5 plasmid has been described (Palma *et al.*, 2005). For BiFC Gateway-compatible pAM-PAT-35S-YFP^N/C^ plasmids were used (Lefebvre *et al.*, 2010). For co-IPs pENTR/D-Topo clones 1-10, 18 and 19 were recombined into pENS-3HA-StrepII (García *et al.*, 2010). His6-tagged *E. coli* expression constructs were generated in pOPIN-F (Berrow *et al.*, 2007). Constructs encoding HaRxL106 (amino acids 46-285), HaRxL106ΔC (amino acids 46-227), HaRxL106ΔC-SV40NLS and ΔIBB-MOS6 (amino acids 66-531) were cloned into pOPIN-F using recombination methods. The pOPIN-F-SAP11 clone (amino acids 32-121) was generated in the lab of Saskia Hogenhout (JIC, Norwich) following the same strategy.

***E. coli protein expression and purification***

Soluble His6-ΔIBB-MOS6 and His6-HaRxL106 were expressed in SoluBL21 cells (Genlantis). His6-HaRxL106ΔC and His6-SAP11 were expressed in SoluBL21 cells transformed with the Rosetta pRARE plasmid. Bacterial cultures were grown to OD_600_ 0.8-1.0 in LB broth at 37 ºC, then cooled to 18 ºC and protein expression was induced by 1mM IPTG followed by overnight incubation at 18 ºC. For His6-HaRxl106, DMSO was added to a final concentration of 0.4% in the growth medium to promote expression of soluble protein. The cells were harvested by centrifugation at 5000 x *g* and cell pellets were frozen at -80 ºC. The pellets were resuspended in 50 mM HEPES, 300 mM NaCl, 25 mM imidazole, pH 7.8 and bacteria were lyzed by addition of lysozyme followed by sonication. Debris and insoluble proteins were cleared by centrifugation (30,000 x *g*, 4 ºC, 20 min) and the supernatant was loaded onto pre-equilibrated Ni^2+^-immobilized metal ion affinity chromatography columns. Following column washing, bound proteins were eluted in 50 mM HEPES, 300 mM NaCl, 250 mM imidazole, pH 7.8 and directly injected on Hi-Load 26/60 Superdex 75 (HaRxL106, HaRxL106ΔC, SAP11) or Superdex 200 (ΔIBB-MOS6) size exclusion columns (GE Healthcare) using 20 mM HEPES, 150 mM NaCl, pH 7.5 as the running buffer. Proteins were concentrated using ultrafiltration columns (Sartorius) and snap-frozen in liquid nitrogen.

***Crystallization of ΔIBB-MOS6 and structure determination***

For crystallization of ΔIBB-MOS6, the N-terminal His6-tag was cleaved by 3C protease and the protein was re-purified by size exclusion chromatography. ΔIBB-MOS6 was concentrated to 12.5 mg/ml and used for crystallization screening. ΔIBB-MOS6 crystals formed in 0.2 M Mg-acetate, 0.1 M Na-cacodylate pH 6.5, 20% PEG8000. Crystallization conditions were further optimized to 0.2 M Mg-acetate, 0.1 M MES pH 6.5, 19% PEG3350 in hanging drop plates. Crystals were harvested in Paratone-N and snap-frozen in liquid nitrogen. A diffraction data set from a single crystal was collected on beamline i02 at Diamond Light Source (Oxford, UK). X-ray data were processed with iMosflm (Leslie, 2006) and scaled with Aimless (Evans and Murshudov, 2013) from the CCP4 suite (Collaborative Computational Project, Number 4, 1994). For X-ray data collection statistics see Table S1. The ΔIBB-MOS6 structure was solved my molecular replacement using Phaser (McCoy *et al.*, 2007) and rice importin-α1a (PDB 4B8J) as a search model. Iterative building and refinement cycles using Coot (Emsley *et al.*, 2010), Refmac5 (Murshudov *et al.*, 2011) and Phenix (Adams *et al.*, 2010) were used to obtain the final model with statistics given in Table S2. Validation tools in Molprobity (Chen *et al.*, 2010) and Coot were used to analyse the final structure. 3D visualizations of protein structures were prepared using PyMOL software v1.7.2 (http://sourceforge.net/projects/pymol/).

***Mass spectrometry***

Samples for LC MS analysis were prepared by excising bands from one-dimensional SDS-PAGE gels stained with colloid Coomassie Brilliant Blue. The gel slices were destained with 50% acetonitrile, and cysteine residues modified by 30 min reduction in 10 mM DTT followed by 20 min alkylation with 55 mM chloroacetamide. After extensive washing with destaining solvent and 100% acetonitrile, gel pieces were incubated with trypsin (Promega) in 100 mM ammonium bicarbonate and 5% acetonitrile in water at 37 ºC overnight.

LCMS/MS analysis was performed using a hybrid mass spectrometer LTQ-Orbitrap XL (ThermoFisher Scientific) and a nanoflow-UHPLC system (nanoAcquity, Waters Corp.) The generated peptides were applied to a reverse phase trap column (Symmetry C18, 5 μm, 180 μm x 20 mm, Waters Corp.) connected to an analytical column (BEH 130 C18, 1.7 μm, 75 μm x 250 mm, Waters Corp.) in vented configuration using nano-T coupling union. Peptides were eluted in a gradient of 3-40 % acetonitrile in 0.1 % formic (solvent B) acid over 50 min followed by gradient of 40-60 % B over 3 min at a flow rate of 250 nL min^-1^ at 40°C. The mass spectrometer was operated in positive ion mode with nano-electrospray ion source with ID 0.02mm fussed silica emitter (New Objective). Voltage +2kV was applied via platinum wire held in PEEK T-shaped coupling union. Transfer capillary temperature was set to 200 ºC, no sheath gas, and the focusing voltages in factory default setting were used. The Orbitrap, MS scan resolution of 60,000 at 400 m/z, range 300 to 2000 m/z was used, and automatic gain control (AGC) target was set to 1000000 counts, and maximum inject time to 1000ms. In the linear ion trap (LTQ), MS/MS spectra were triggered with data dependent acquisition method for the 5 most intense ions. The threshold for collision-induced dissociation (CID) was above 1000 counts, using normal scan rate types, AGC accumulation target was set to 30,000 counts, and maximum inject time to 150ms. A data dependent algorithm was used to collect as many tandem spectra as possible from all masses detected in master scan in the Orbitrap. For the latter, Orbitrap pre-scan functionality, isolation width 2 *m/z* and collision energy set to 35 % were used. The selected ions were then fragmented in the ion trap using CID. Dynamic exclusion was enabled allowing for 1 repeat only, with a 60 sec exclusion time, and maximal size of dynamic exclusion list 500 items. Chromatography function to trigger MS/MS event close to the peak summit was used with correlation set to 0.9, and expected peak width 7s. Charge state screening enabled allowed only higher than 2+ charge states to be selected for MS/MS fragmentation.

***Software processing and peptide identification***

Peak lists in format of Mascot generic files were prepared from raw data using Proteome Discoverer v1.2 (ThermoFisher Scientific) and concatenated using in house developed Perl script. Peak picking settings were as follows: m/z range set to 300-5000, minimum number of peaks in a spectrum was set to 1, S/N threshold for Orbitrap spectra set to 1.5, and automatic treatment of unrecognized charge states was used. Peak lists were searched on Mascot server v.2.4.1 (Matrix Science) against TAIR (version 10) database. Tryptic peptides only, up to 2 possible miscleavages and charge states +2, +3, +4, were allowed in the search. The following modifications were included in the search: oxidized methionine (variable), carbamidomethylated cysteine (static). Data were searched with a monoisotopic precursor and fragment ions mass tolerance 10ppm and 0.8Da respectively. Mascot results were combined in Scaffold v. 4 (Proteome Software, (Searle, 2010)) and exported in Excel (Microsoft Office)*.* Peptide identifications were accepted if they could be established at greater than 95.0% probability by the Peptide Prophet algorithm (Keller *et al.*, 2002) with Scaffold delta-mass correction. Protein identifications were accepted if they could be established at greater than 99.0% probability and contained at least 2 identified peptides. Protein probabilities were assigned by the Protein Prophet algorithm (Nesvizhskii *et al.*, 2003). Proteins that contained similar peptides and could not be differentiated based on MS/MS analysis alone were grouped to satisfy the principles of parsimony.
