# Supplementary material for: Probing formation of cargo/importin-α transport complexes in plant cells using a pathogen effector
Source: Plant J. 2014 Nov 17;81(1):40–52. doi: 10.1111/tpj.12691 (PMC4350430; doi:10.1111/tpj.12691)
Supplement: Supplementary file 11 [file tpj0081-0040-sd11.docx]

**Figure S1.** RFP-HaRxL106 is entirely nuclear localized when expressed as transgene in *Arabidopsis thaliana*. Confocal images of p35S-driven RFP-HaRxL106 or a free RFP control construct were taken from the epidermal cell layer of rosette leaves. Localizations are representative of two independent transgenic lines.

**Figure S2.** HaRxL106 amino acids 228-285 are sufficient for MOS6 binding. A series of N-terminal deletion constructs was designed based on the predicted HaRxL106 secondary structure. MOS6-GFP was transiently co-expressed with the HS-tagged HaRxL106 fragments in *N. benthamiana.* 48h post infiltration HaRxL106 fragments were IP-ed and co-purifying MOS6-GFP was detected by an α-GFP Western blot. Upper panel shows total protein extracts and lower panel shows IP samples. The HaRxL106 (228-285) protein was only detectable after IP. NS = non-specific signal of the α-HA antibody following IP.

**Figure S3.** *mos6* mutants are more susceptible to *P. syringae* strains with reduced effector repertoires. Bacterial titres of *P. syringae* DC3000 ΔCEL **(a)** or *P. syringae* DC3000 ΔAvrPto/AvrPtoB **(b)** at the day of infiltration (white bars) and 3 days post inoculation (black bars) on Col-0 and *mos6* mutants. *snc1* and *eds1* are resistant and susceptible controls, respectively. Error bars show standard deviation and asterisks indicate statistical significance (t-test, α=0.05; n=3). Two additional biological replicates of these experiments gave similar results.

**Figure S4.** Sequence alignment between HaRxL106 amino acids 232-279 and Arabidopsis bZIP5 amino acids 61-120. The alignment was generated with ClustalW (Thompson *et al.*, 1994).

**Table S1.** Stochiometry, ΔH and ΔS values for ITC experiments shown in Figure 2c.

**Table S2.** X-ray data collection, refinement, and validation statistics.

**Supporting data S1.** Proteins identified by LC-MS/MS in immuno-precipitates of YFP-HaRxL106 transgenics and controls.

**Supporting data S2.** Maxima of best Mascot ion scores and total spectrum counts for peptides identified by LC-MS/MS.

**Supporting data S3.** List of oligo-nucleotides and pENTR plasmids used in this study.

**Supporting experimental procedures S1.**
